# A new model of tethered cord syndrome produced by slow traction

**DOI:** 10.1038/srep09116

**Published:** 2015-03-13

**Authors:** Sheng-Li Huang, Jun Peng, Guo-Lian Yuan, Xiao-Yan Ding, Xi-Jing He, Bin-Shang Lan

**Affiliations:** 1 Department of Orthopaedics, the Second Affiliated Hospital, School of Medicine, Xi'an Jiaotong University, Xi'an 710004, China; 2 Department of Orthopaedics, Shajing Hospital Affiliated to Guangzhou Medical University, Shenzhen 518104, china; 3 Central Laboratory for Scientific Research, the Second Affiliated Hospital, School of Medicine, Xi'an Jiaotong University, Xi'an 710004, China; 4 Department of Pathogenic Biology and Immunology, School of Medicine, Xi'an Jiaotong University, Xi'an 710061, China

## Abstract

The development of a suitable animal model is important for clarifying the pathogenesis of tethered cord syndrome (TCS). This study was undertaken to develop a new animal model for investigating the pathogenesis and therapeutic strategies for TCS. A traction device, a filum terminale tractor, was designed exclusively for this experiment. A TCS model was produced in cats using the tractor to fixate the filum terminale to the dorsal aspect of the second sacrum. The responses to tethering were evaluated by electron microscopy and electromyography for detection of somatosensory evoked potentials (SEPs) and motor evoked potentials (MEPs) at designated time points. Progressive swaying gait and lameness in clinical performance were observed with cord traction. Histopathological examination revealed an association between the increasing traction in the spinal cord and the increase in impaired nerve cells. No changes of SEPs and MEPs were detected in the untethered cats, while the latencies of SEPs and MEPs significantly increased in the tethered cats. The TCS model established in this study is simple and reproducible, in which varying degrees of tension could be applied to the neural elements.

Tethered cord syndrome (TCS) is a stretch-induced functional disorder of the lumbosacral spinal cord due to excessive tension that develops usually between two fixation points^1^. The cord is abnormally fixed to a caudally immovable structure of the spine that limits motion of the cord in the caudal-rostral direction. The lesion is caused by congenital or acquired factors^2^,^3^,^4^,^5^,^6^,^7^ leading to the tethering of the distal part of the spinal cord, which may result in neurological, musculoskeletal, urological, or gastrointestinal abnormalities^8^.

While TCS is a clinical entity, the pathophysiology of the lesion is not clear, and it remains controversial whether and when the tethered cord should be surgically corrected. Suitable animal models are crucial for classifying the pathogenesis of the disease. However, to date there has been a paucity of experimental models used for the investigation of this problem. It is therefore highly desirable that an appropriate animal model be established for studying the underlying pathophysiologic mechanisms of TCS and to develop intervention strategies for this lesion. There are two classical inductive protocols commonly used for establishing TCS cats relevant to spinal cord traction. One is acute traction^9^, composed of firstly isotonic traction with weights and subsequently isometric stretching by a stitch to the dura mater. The other is chronic traction^10^, which induces long-term effect of isometric cord stretching, but accompanied with symptomatic fluctuation. In children with TCS, rostral ascension of conus medullaris is prevented as the child grows because of the mechanical traction caused by intradural abnormalities. Cord tension may then increase as the spine grows along with the child's normal growth. It is an important prerequisite for TCS study to establish a simple and pragmatic animal model which can simulate this progressive process. Currently, investigation of the pathophysiology and further the intervention strategies of TCS are limited by the absence of an ideal animal model. An ideal model should allow for dynamic traction in duration of weeks or months, which may further lead to variations in traction power. However, the stretching of the spinal cord produced by acute experimental tethering could not simulate TCS in human. The chronic stretching, although in a manner similar to human TCS, is not a constant dynamic stretching. To overcome these problems, the present study attempted to refine the methods for animal modelling in TCS study.

## Results

### Clinical status of the animals

There were no wound dehiscence and wound infection. All cats showed reduced activity and reluctance to jump immediately after operation, but recovered 2 days later from the procedure. During the first two days after operation, mild discomfort was elicited on palpation of the lumbar spine.

Cats in the three traction groups could walk at week 2 of the cord traction. But they displayed a lameness of the pelvic limb and a swaying gait. The swaying gait was subtle and presented only in quick turn. With the lengthening of the cord traction, the hind limbs became weakened. After the animals underwent 4 weeks of traction, they could stand, but 8 out of 10 cats presented the reluctance to rise from a sitting or lying position. At week 6, hind limb weakness and gait disturbance exacerbated. The cats were unable to stand even when helped. These results indicated an association of progressive neurological deterioration with continued traction of the spinal cord.

### Histopathological findings

**Figure 1:**
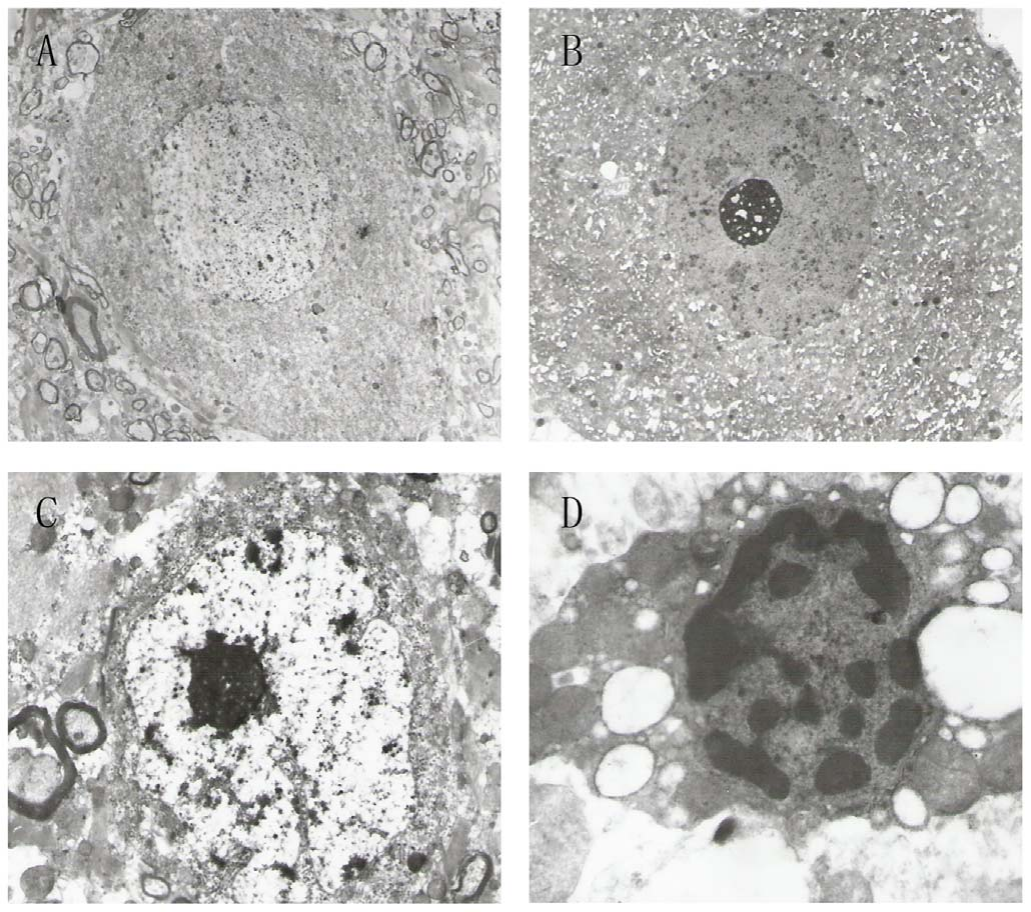
Electron microscopy findings. (A): Neurons were normal before traction (×2000). (B): Edema was observed in neurons 2 weeks after traction (×2000). (C): Neurons showed nuclear membrane shrinkage 4 weeks after traction (×5000). (D): Neurons showed chromatin margination in the nucleus 6 weeks after traction (×10000).

There was no deep infection when the cats were euthanized. Histopathological examinations of the cats appeared normal before the traction was performed. After traction, different degrees of neuronal damage were seen in the gray matter. The stretching of the cord induced ultrastructural changes in neurons, including edema, loss of organelles (rough endoplasmic reticulum), loss of cristae in mitochondria, chromatin margination in the nucleus, and pronounced vacuolization. In the white matter, there were large vacuoles and degenerated myelin bodies. Definite histological changes were noted after 2 weeks of traction, and the changes developed to be more marked after 6 weeks (Figure 1). Our data suggested that the histopathological changes were proportional to the amount of caudal traction applied to the distal cord.

### Electrophysiological findings

Somatosensory evoked potentials (SEPs) and motor evoked potentials (MEPs) in all the groups did not show significant changes in latency immediately after operation. In Group 1, before operation the latency of SEPs and MEPs ranged from 6.0 to 6.5 milliseconds (ms) and from 2.7 to 3.1 ms, respectively; after operation they ranged from 5.7 to 6.9 ms and from 2.6 to 3.2 ms. There was no difference in the latencies before and after operation in Group 1(p > 0.05). In Group 2, after 0.01 Newtons (1 g weight) traction, the latency of SEPs and MEPs ranged from 6.0 to 7.1 ms and from 2.6 to 3.1 ms. There was no difference between the latencies before operation and after 0.01 Newtons traction (p > 0.05) (Table 1). These results verified that the spinal cord would not be damaged during the operation. Two weeks following the tethered cord formation, the latency of SEPs and MEPs ranged from 7.1 to 8.5 ms and from 3.3 to 3.7 ms, respectively. However, four weeks after tethering they were found to be ranging from 8.7 to 9.7 ms and from 3.5 to 4.0 ms. Six weeks following the tethering, the latencies ranged from 10.1 to 11.6 ms and from 3.8 to 4.4 ms, respectively. The elongation of the latent period could be observed in all the three traction groups (p < 0.05) (Table 1). Latency increases were consistent in all animals. There were significant changes in latency among weeks 2, 4 and 6 post tethering (p < 0.01).

## Discussion

A TCS animal model in cats was established in the present study, in which the pathophysiological changes were created by longitudinal traction on the spinal cord. Experimentally, the model shows a parallel between the extent of elongation of the spinal cord and the degree of the deterioration of evoked potential activity, and these are related to clinical manifestation and histopathological changes. Further, the model also provides us detailed neuropathologic information of TCS. The high incidence of tethering and no mortality in cats treated with this inductive protocol indicate its applicability to future studies. In summary, this experiment provides a method of TCS modelling, which paves a way for future research on the potential treatment strategies of this disease. The model reported in this article is a reliably reproducible model of TCS, which could be a safe and effective alternative to traditional methods.

TCS is a progressively debilitating neurological condition caused by abnormal longitudinal traction on the caudal spinal cord. A number of experimental models have been developed for evaluating the pathophysiology of this disease. However, in the majority of existing experimental animal models, longitudinal traction is induced by hanging weights^9^,^11^,^12^, and the accuracy and feasibility of the models thus remain controversial. Because of the lack of appropriate experimental models, little preclinical research on the pathogenic process of TCS has been reported.

In children, the tethering of the filum terminale is placed under tension as the child grows and symptoms develop. In an experimental cat model, a progressive amount of traction is placed on the filum terminale in a controlled setting. Periodic electrophysiologic tests, as well as pathological and neurologic examinations, are necessary for an accurate judgment of whether the spinal cord will be damaged or not by acute traction, and to check the degree of recovery after detethering. In addition, complete untethering may not be achieved if short sacral nerve roots do not allow full mobilization of the spinal cord. However, the model developed in this study may help reduce traction injury to nerve roots. It is very difficult to develop an accurate quantitative, chronic injury model for TCS. In the models of chronic tethering by stitch to the dura mater, it has not been possible to correctly measure the degree of traction on conus medullaris. To replicate the physiological condition of the disease, we applied 1 g weight of tension to the terminal filum. According to a previously reported animal study^9^, a weight higher than this could cause damage to the spinal cord.

Most researchers have adopted spinal cord traction procedure that fixes the filum terminale to dura mater. However, it is not easy to quantify the traction force applied to the spinal cord with this method. For this reason, we have designed a filum terminale tractor which can exert various degrees of traction. Because of significant individual differences in the size of the spinal canal, the degree of conus medullaris traction cannot be directly expressed by the length of traction. The duration of traction is therefore adopted as an indicator of the traction degree. In our experiments, the cats underwent different durations of stretching which induced different degrees of traction, and the effects of these different tractions could thus be compared. The spinal cord might have been damaged gradually but severely if the cord had been continually stretched through the motion of the disc in the tractor. We postulate that the longer duration of the spinal cord traction is, the greater the tensile force in the cord tissue will be. If the lumbosacral cord suffers from excessive tensile force by traction, spinal cord function will be impaired. The study reported here defines a cat model of TCS which would take several weeks to develop and produce consistent pathophysiological changes similar to those in human. In addition, the model is highly reproducible.

Since the process of TCS is a progressive one, the course of clinical presentation is related to the degree of traction and not necessarily its cause^8^. Traction and elasticity of the spinal cord are fundamental factors underlying the pathophysiology of TCS^13^, and the conus medullaris is the region most vulnerable to traction^12^. We identify the degree of cord traction rather than the level of tethering as the predominant factor related to the onset of symptoms. In our experiment, the histopathology of the cat spinal cord was assessed by using our self-designed tractor under various mechanical tension scenarios. SEPs which were used in parallel to monitor the electrophysiological changes demonstrated patterns consistent with the histopathological findings. The results suggest that local traction contributes to the pathophysiological progression of TCS. Therefore, the degree of traction is an important factor in the evaluation of the lesion.

This study had the following limitations. First, although the pathophysiology of TCS could be induced from the stretching effects on the spinal cord, the natural history of the disease cannot be imitated. This is because the conus medullaris migrates toward the coccyx in the model, but in human the conus medullaris is restricted to ascend. Second, the animal model established here might not possess the same biomechanical properties of human in neural tissue.

In conclusion, the presented model may provide reliable experimental conditions of TCS with reproducible neurophysiologic and morphologic effects. The model allows us to better understand the pathophysiology of TCS, and potentially to develop and evaluate the effects of various therapies. Further research will be necessary to understand the biochemistry changes of TCS. The model would also be suitable for studies of spinal cord compression under TCS.

## Methods

### A filum terminale tractor

**Figure 2:**
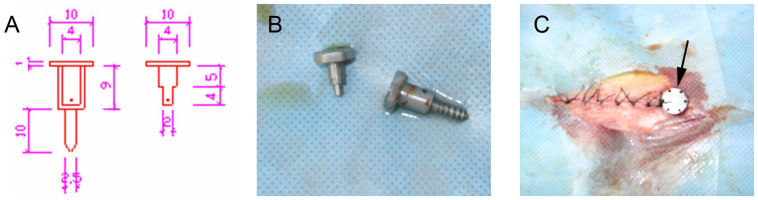
Self-designed filum terminale tractor. (A): Schematic diagram of the tractor (measurement unit: millimeter). (B): Photograph of the tractor. (C): Photograph of the tractor placed at the second sacral vertebra.

A filum terminale tractor was designed in this study based on the fact that the caudal spinal cord is restricted in vertical movement and increased cord tension is accentuated during rapid growth of the spine (Fig. 2A, B). The tractor, made entirely of titanium, consists of two cylinders. The outer cylinder is 9 mm long with an inner diameter of 4 mm, a wall thickness of 1 mm, a ring-shaped disc with a diameter of 10 mm attached transversely to the cylinder axis at one end, and a screw 10 mm long with a diameter of 2.5 mm attached parallel to the cylinder axis at the other end. The inner cylinder is constituted by a large cylinder 5 mm long with an inner diameter of 4 mm, a small cylinder 4 mm long and 2 mm wide, and a round disc with a 10 mm diameter attached transversely to the axis of the large cylinder at one end. The outer and inner cylinders comprise respectively circular holes of 0.3–0.4 mm in diameter, in which a silk thread can be inserted through. Six small holes evenly distribute on the circumference of each disc. Through one hole in each disc, two L-shaped stainless pins are inserted. The scale of the disc rotation from a hole to another is 1 mm.

To mimic TCS, the filium terminale was tethered to the tractor. A silk thread was threaded through the outer cylinder, one end attached to the inner small cylinder while the other tied around the filium terminale at a location 5 mm below the conus medullaris.

### Animal grouping

Principles of laboratory animal care were followed and all procedures were conducted according to the guidelines established by the National Institutes of Health, and every effort was made to minimize suffering. This study was approved by the Animal Experiment Committee of Xi'an Jiaotong University.

Twenty-five adult male domestic cats, body length (anal-nasal) 40–50 cm, were used in our experiments. The cats were divided into five groups with five cats in each group. Group 1 was set as control group of five cats in which the filum terminale was stretched by 0.01 Newtons. Electrophysiology monitoring was performed, followed by immediate euthanasia for spinal cord specimens. Group 2 was a sham traction group, in which the filum terminale tractor was inserted at the second sacral vertebra and placed for 4 weeks without traction. Group 3 was the mild traction group, where cats were treated as in Group 2, but followed with traction 3 days after the insertion of the tractor. The traction was performed by rotating the disc for one scale (1 mm) every 3–4 days (2 scales per week) for 2 weeks. The animals were then euthanized by an injection of ketamine and exsanguination for the subsequent examinations. Group 4 and Group 5 were set as the moderate and strong traction groups, in which cats were treated similarly as those in Group 3, except that traction was performed for 4 and 6 weeks, respectively.

### Treatment

**Figure 3:**
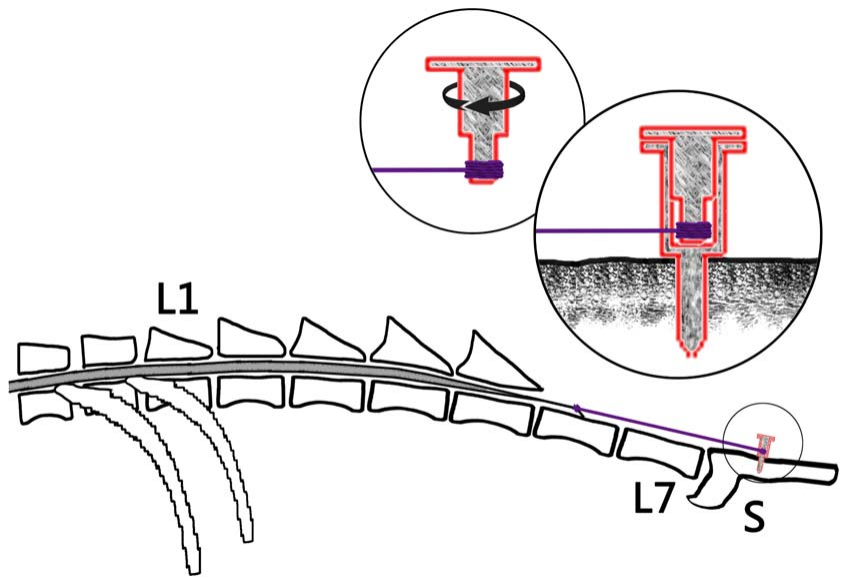
Diagram showing the filum terminale stretched by tractor.

All surgical procedures were performed under aseptic conditions. The cats were anesthetized with intramuscular injection of ketamine at 30–40 mg/kg body weight. After anesthetization, the cat was positioned in sternal recumbency and both pelvic limbs were kept fully flexed close to the body wall. After routine aseptic preparation, a standard dorsal midline approach was performed to extend the vertebral body of the fifth lumbar vertebra (L5) to the third sacral vertebra (S3). The dorsal spinous processes of L5 through S3 were removed with rongeurs. A dorsal laminectomy, preserving the articular facet joints, was performed to expose the dural sac. A 10 mm long screw in the filum terminale tractor was inserted dorsoventrally into the mid-body of S2. The screw through the vertebral body was placed in such a way to avoid injury of the nerve roots. A midline durotomy was performed, and the filium terminale was isolated. In order to maintain the tension on the neural tissues, one end of a 2-0 silk ligature was tied around the filium terminale and the ligation was positioned 8 mm caudal to the lowest end of the cord; the other end of the ligatures passed over a pulley and attached to a weight of 0.01 Newtons. The height of the pulley became straight line to the long axis of the lumbosacral cord in the direction of the traction. Then a 1-0 silk suture from the tractor was tied rostral to the ligature knot while traction was maintained. The filium terminale was sectioned between the ligature knots with upper section 6 mm below the conus-filum junction, and the filium terminale was transected at the distal end above dural sheath. Strong manipulation was avoided to prevent injury to the spinal cord. The dura was then closed, and a thin free fat graft from the subcutaneous tissue was applied to cover the laminectomy site. The incision was closed in layers and antibiotic ointment was applied. The animals received postsurgical analgesic (butorphanol tartrate, 0.3 mg/kg for 6 h) as needed and a broad-spectrum prophylactic antibiotic agent (Cefazolin, 150 milligram, intramuscular injection) was administered daily for 3 days. The disc was left protruding from the skin surface (Fig. 2C). To prevent the filum terminale being cut off by the traction force, rubber tissue was located superficially on the filum terminale before the ligature knot was tied. The traction was gradually increased by rotating the disc of the inner cylinder through stepwise increments of 1 mm at determined time intervals. Hereby, varying degree of spinal cord traction was achieved by the self-designed tractor (Fig. 3).

For the control group, operation only and placement of tractor but no traction was done.

### Electromyography

Since the tethered cord is characterized by suppression of electrophysiological activities^9^,^14^, electromyography was performed to monitor the changes in the activities. SEPs and MEPs in all the cats were examined and recorded before and after surgery, and before euthanasia as well. Because SEPs measure mainly the sensory nerve transmitting path, which passes along the dorsal part of the spinal cord and reflects the function of the posterior funiculus of the spinal cord mainly, and MEPs measure mainly the motor nerve transmitting path, which passes along the ventral part of the spinal cord, combination of SEPs and MEPs have been used increasingly to monitor the extent of spinal cord injury. The time from stimulation to the first wave crest was referred to as latency. The values of latencies in all the groups were expressed in ms. The post-operative electrophysiology served as a baseline for traction. Changes of latency were analyzed in relation to the course of traction.

For the monitoring of SEPs, the recording and reference electrodes were placed on the vertebral plate approximately 10 cm cranial to the spina iliace level. A bipolar nerve-stimulating electrode (electrode points 2.5 cm apart) was placed subcutaneously along the left posterior tibial nerve. A series of single-phase current stimuli (500 responses) with an intensity of 3.0 mA, mean frequency of 2 Hz and duration of 1.0 ms, was generated by a stimulator. To detect MEPs, a stimulating electrode was placed on the vertebral plate approximately 10 cm cranial to the spina iliace level, while a recording electrode was placed in the gastrocnemius muscle.

There existed risk of damaging the spinal cord while performing this manipulation, so intraoperative electromyography was used to distinguish the nerve roots from the filum.

### Histopathology

After the prescribed period of traction, intraventricular perfusion of ice-cold normal saline was performed in the animals to remove the blood till the lungs turned white. The animals were then fixed with a mixture of 4% paraformaldehyde and 1% glutaraldehyde in 0.1 mol/L phosphate buffer. Since the lumbosacral segment of the spinal cord is the most affected area in TCS, the conus medullaris was removed and fixed by immersion in a mixture of 2% paraformaldehyde and 2.5% glutaraldehyde. Post-fixation was carried out with 1% osmium tetroxide, and dehydration was carried out in a series of graded ethanol. Then, the tissue was embedded in Epoxy resin. Ultrathin sections were cut with a microtome with the thickness of 100 nm and stained with uranyl acetate followed by lead citrate, and analyzed and photographed under a transmission electron microscope.

### Data analysis

Data were expressed as mean ± SD, and were analyzed using SPSS 16.0. Wilcoxon Signed-Rank test was performed to evaluate the change of electrophysiology pre- and post-operation or traction. Kruskal-Wallis test was used to compare data among the three traction groups. P < 0.05 was considered as statistically significant.

## Author Contributions

S.L.H. designed the study and drafted the manuscript; J.P. performed animal experiment and statistical analysis; G.L.Y., X.Y.D. and X.J.H. performed animal experiment; B.S.L. conceived and designed the study. All authors reviewed the final version.

## Figures and Tables

**Table 1:** Latency changes of every groups(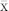 ± s, ms)

				Traction groups		
	Preoperation	Postoperation	Sham traction group (4 weeks)	2 weeks	4 weeks	6 weeks
SEP	6.28 ± 0.22	6.36 ± 0.43	6.52 ± 0.41	7.76 ± 0.61	9.12 ± 0.40	10.88 ± 0.55
MEP	2.90 ± 0.16	2.88 ± 0.25	2.84 ± 0.23	3.50 ± 0.19	3.70 ± 0.20	4.10 ± 0.22
